# Trans-sacral bar osteosynthesis provides low mortality and high mobility in patients with fragility fractures of the pelvis

**DOI:** 10.1038/s41598-021-93559-0

**Published:** 2021-07-09

**Authors:** Daniel Wagner, Miha Kisilak, Geoffrey Porcheron, Sven Krämer, Isabella Mehling, Alexander Hofmann, Pol M. Rommens

**Affiliations:** 1 Department of Orthopaedics and Traumatology, University Medical Center Mainz, Langenbeckstr. 1, 55131 Mainz, Germany; 2 Department of Traumatology, University Medical Centre Ljubljana, Ljubljana, Slovenia; 3 Division of Hand Surgery, St. Vincent Hospital Hanau, Hanau, Germany; 4 Department of Orthopaedics and Traumatology, Westpfalz-Klinikum Kaiserslautern, Kaiserslautern, Germany

**Keywords:** Outcomes research, Trauma, Osteoporosis

## Abstract

Operative treatment of osteoporosis-associated fragility fractures of the pelvis (FFP) and the sacrum is advocated with immobilizing or longstanding pain, fracture progression and displacement. We analyzed clinical outcomes regarding mobility, quality of life, and mortality of patients with FFP treated with trans-sacral bar (TB) osteosynthesis through S1. Demographics, clinical data, and operation-related data of patients with an FFP treated with TB were acquired from chart review. We assessed mortality, quality of life (EQ-5D), mobility, and residential status at follow-up. Seventy-nine females and six males with a median age of 78.0 years (IQR 73–84) were included, median follow-up was 3.2 years. Medical complications during hospitalization occurred in 28%. Operative revision was carried out in 15% of patients. One-year survival was 90.4%, this was associated with shorter preoperative and total length of stay in hospital (p 0.006 and 0.025, respectively). At follow-up, 85% lived at home and 82% walked with or without walking aid. Higher EQ-5D was reached with higher mobility status and living at home (p < 0.001 and < 0.001, respectively). TB osteosynthesis is an adequate and reliable method for fixation of FFP in the posterior pelvic ring to ensure timely mobilization. Shorter preoperative and total length of stay had lower mortality rates, advocating a standardized management protocol to limit time delay to operative therapy. Patients treated with TB osteosynthesis had low 1-year mortality of less than 10%.

## Introduction

Fragility fractures of the pelvis (FFP) often lead to decreased mobility^1^, loss of independence and institutionalization^2^. Especially patients undergoing surgery had delayed presentation with a median of 14 days^3^. There is an excessive mortality^4^ with a 1-year mortality is as high as 17–30%^1,4–6^, similar to the 1-year mortality in hip fractures in elderly (19–33%)^7,8^. Surgically treated patients with an FFP had a lower mortality^5^.

Fractures of the posterior pelvic ring are found in 82% of FFP, mostly localized in the sacrum^9^. Surgical fixation of sacral fractures is usually performed by minimal-invasive iliosacral (IS) screws. Due to decreased bone mass in elderly and patients with a fragility fracture of the sacrum^10,11^, implant loosening with backing out is seen in 14–20% with 18% necessitating operative revision^12,13^. Alternatively, trans-sacral bar (TB) using washer and nuts on both sides^14^, augmented IS screws^15^, transiliac internal fixator^16^, or spinopelvic stabilization are used. Lower rates of implant loosening have been demonstrated when using trans-sacral implants^13^, confirmed by higher biomechanical strength compared to bilateral IS screws^17^. The shape and size of the upper sacrum limit safe trans-sacral implant positioning in half of patients^18,19^.

We retrospectively studied patients suffering from an FFP treated with minimal-invasive TB osteosynthesis through S1. By assessing medical and implant-related complications as well as mortality and functional outcome, our assumption was that TB osteosynthesis is a reliable and stable osteosynthesis of the posterior pelvic ring which lowers mortality due to better mobilisation.

## Materials and methods

**Figure 1:**
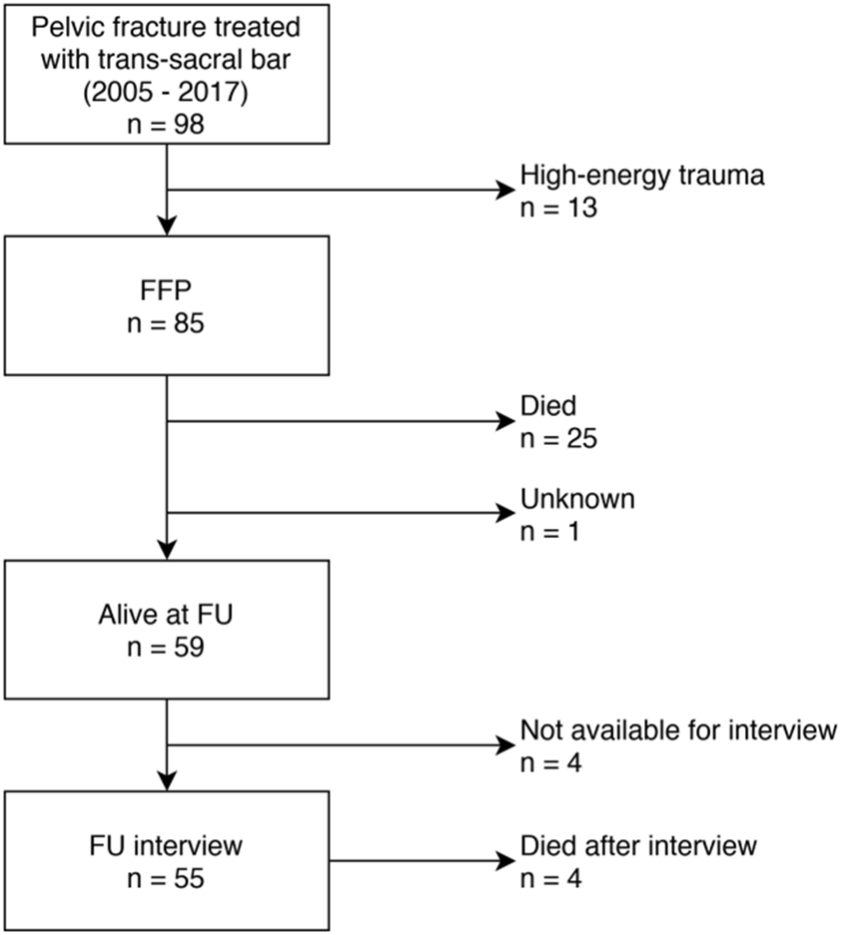
Flow diagram with patient inclusion and follow-up. *FFP* fragility fracture of the pelvis, *FU* follow-up. Graphic produced with draw.io (accessed 10.04.2020).

We retrospectively reviewed medical charts of adult patients with a pelvic fracture after low-energy trauma admitted at our center from 2005 to 2017. Low-energy fractures of the posterior pelvic ring were stabilized in 85 patients with TB (Fig. 1). Demographic and surgery-related data were collected: FFP classification according to Rommens and Hofmann^9^, operative details, length of stay (LOS), medical and implant-related complications, American Society of Anesthesiologists (ASA) physical status, medication, and following comorbidities: dementia, diabetes mellitus, cardiovascular disease, pulmonary disease, rheumatoid arthritis, and known malignancy. Symptomatic medical complications included: deep venous thrombosis, pulmonary embolism, cardiovascular events, urinary tract infections, pneumonia, and skin ulcer. Delayed presentation was defined as ≥ 7 days after trauma. Images were assessed for hip replacement or lumbar spine fusion.

### Treatment protocol

**Figure 2:**
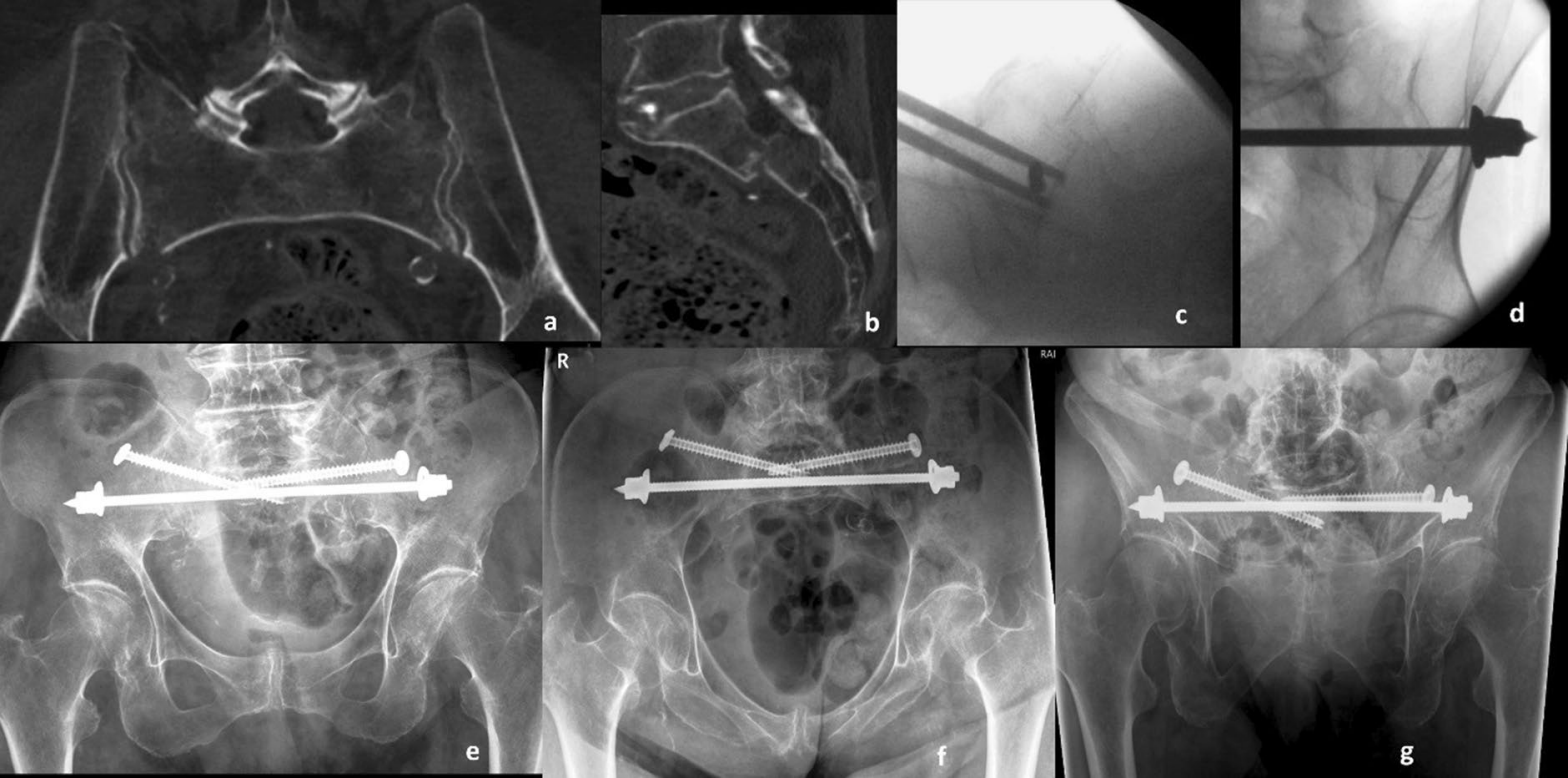
92 year old female patient presenting 1 week after fall from standing height with immobilizing pain at lower lumbar spine. (**a**) Axial CT demonstrating slightly displacement of bilateral sacral fracture; (**b**) sagittal CT with displaced transversal fracture at S1/S2 level. The fracture was classified as FFP type IVb. (**c**) Lateral fluoroscopic image orientated in direction of the patient (bottom anterior, left cranial) showing overlapping of iliocortical densities and ischial notch. The drill guide is placed centrally in S1 corridor caudal to the iliocortical densities. (**d**) Oblique obturator view to assess the abutment of the washer on the iliac cortex. Postoperative control at discharge, patient is mobile with a walker ((**e**) anterior–posterior, (**f**) inlet, and (**g**) outlet view).

Patients with a FFP are admitted after complete diagnostics with conventional radiograph and CT scan. Isolated anterior fractures (FFP type 1) and non-displaced posterior lesions (FFP type 2) are treated conservatively with analgesics and physiotherapy-assisted mobilization. If these patients suffer immobilizing pain or prolonged pain without adequate mobilization after 5 to 7 days or if patients present posterior displaced fractures (FFP types 3 and 4, Fig. 2a,b), we recommend operative stabilization.

### Trans-sacral bar osteosynthesis

Similar to IS screw, the TB crosses the vertical sacral fracture perpendicularly. Tightening of the nuts exerts slight interfragmentary compression. It is blocked bilaterally with locknuts; thus TB can be used in uni- and bilateral sacral fractures. The implant is load sharing, positioned in the center of weight transmission in S1: it transfers load from the spine to the posterior ilium. The strength of TB does not depend on the screw purchase in the reduced bone mass of the sacrum^10,11^, but on cortical bone of the posterior ilium.

Preoperative planning is mandatory^19^: the corridor’s dimension is evaluated in a coronal and axial view (according to the axis of the sacrum). Trans-sacral fixation should be avoided in dysmorphic sacra lacking safe space, the corridor’s dimension should be at least twice the TB’s diameter^18^. In these cases, a transiliac internal fixator or spinopelvic fixation is used. Bowel is prepped with purgatives the day before surgery for better intraoperative visualization.

The patient is placed prone on a radiolucent table. Fracture reduction is not carried out in posterior lesions of osteoporotic FFP. A strict lateral view is obtained using fluoroscopy, confirmed by overlapping of the sciatic notches and the iliocortical densities (ICD). The fluoroscopic image is orientated in the same direction as the patient (Fig. 2c). The S1 corridor is located caudally to the ICD, which marks the inner cortical layer of the iliac fossa near the IS joint. Subcutaneous tissue and muscles are split bluntly after skin incision. A 2.8 mm guide wire with drill bit is positioned centrally in the S1 corridor. With slight mallet blows, the first cortex is perforated. Using inlet and outlet projection, correct positioning is controlled and drilling is continued until it becomes palpable on the contralateral side. The anteroposterior position is confirmed with inlet view, the outlet view is used to assure position above the S1 sacral foramina. After contralateral skin incision the wire is held with a clamp. The wire is overdrilled with a 4.5 mm cannulated drill, leaving the 2.8 mm wire in place. A 6.0 mm non-cannulated sacral bar (Depuy Synthes, Oberdorf, Switzerland) mounted on a power drill is inserted in the prepared 4.5 mm canal. Under fluoroscopic control, the bar is inserted from one side to the other, while retracting the 2.8 mm drill from the other side tapping slightly against the tip of the sacral bar (kissing technique). After a washer and two nuts are placed and tightened contralaterally, the bar is retracted using backward drilling until the washer hits the lateral cortex of the ilium visible in the fluoroscope—tilted perpendicular to the posterior ilium (Fig. 2d). A washer and two nuts are inserted and slightly tightened. The overlapping part of the bar is cut with a bolt cutter. The TB is augmented with an IS screw on the fractured side to increase rotational stability. In cases of additional anterior stabilization, the patient is turned to supine position and again prepped. Conventional intraoperative fluoroscopy without navigation or 3D-imaging was used due to good experience in posterior pelvic fixation^20^. If possible, patients are mobilized with weight-bearing as tolerated. Routine postoperative imaging consisted of conventional X-rays with anteroposterior, inlet, and outlet view (Fig. 2e,f).

### Follow-up (FU)

Patients or their relatives were contacted by phone^21^. If not possible, their general practitioner or the bureau of vital statistics was contacted to ask about vital status. Quality of life (QoL) was assessed by EuroQol 5D (EQ-5D) 5 level questionnaire, scores range from − 0.205 to 1.0 (higher indicates better QoL)^22^. The actual place of living and mobility status was asked, the mobility specified by Parker Mobility Score (PMS), ranging from 0 to 9 (higher indicates better mobility)^23^. Numeric pain score (NRS) and subsequent osteoporotic fractures were questioned.

### Statistics

Normal distribution was assessed with Kolmogorov–Smirnov test. Descriptive statistics were described as mean and standard deviation in normally distributed data and as median and interquartile range (IQR) for non-normally distributed data. Groups were compared using the nonpaired Students t-test (normally distributed data) and the Mann–Whitney-U test (non-normally distributed data). Nominal groups were compared using the chi-square test, odds ratio (OR) and 95% confidence interval (CI) was calculated. Survival analysis was computed according to Kaplan–Meier. A p-value of ≤ 0.05 was considered significant. Statistical analysis was performed using SPSS software (IBM SPSS Statistics, Version 23; IBM Corp, Armonk, NY, USA).

### Ethics

Approval for this retrospective study has been granted by the local ethics committee (837.140.17 (10974); Ethics Commission of the State Chamber of Medicine of Rhineland-Palatinate, Germany). Patients confirmed voluntary participation with the study, informed consent was obtained by living patients or their legal guardian. This study was conducted in accordance with Good Clinical Practice Guidelines.

## Results

We included 85 patients treated with TB osteosynthesis (Table 1). Seventy-nine were females (92.9%) and six males (7.1%). The median age was 78.0 years (IQR 73–84, 50–95; median for males 85, and females 77, p 0.358). Time from trauma or begin of symptoms to presentation was at median 15 days (IQR 4–81, 0–863), two patients could not specify.

**Table 1:** Fracture-related information and medical history.

	Number (total = 85)	Percentage	
**Trauma mechanism**			
Fall from standing height	53	63%	
No trauma memorable	30	35%	
Recurrent falls	2	2%	
**FFP classification**			
FFP 2 (posterior non-displaced)	31	36%	
2a			6
2b			16
2c			9
FFP3 (posterior unilateral displaced, all 3c)	3	4%	
FFP4 (posterior bilateral displaced)	51	60%	
4b			46
4c(1)			5
**Concomitant fracture anterior pelvis**			
None	22	26%	
Unilateral	45	53%	
Bilateral	18	21%	
**ASA**			
ASA 2	24	28%	
ASA 3	57	67%	
ASA 4	4	5%	
**Comorbidities**			
Dementia	6	7%	
Cardiovascular disease	68	80%	
Pulmonary disease	11	13%	
Diabetes mellitus	13	15%	
Rheumatoid arthritis	6	7%	
Known malignancy	25	29%	
Preexisting osteoporosis	63	74%	
**Number or comorbidities (2)**			
0	7	8%	
1	38	45%	
2	29	34%	
3	11	13%	
**Medication**			
Corticosteroids	8	9%	
Anticoagulation	15	18%	
Antithrombotics	21	25%	
Previous hip replacement	17	20%	
Previous lumbar fusion	4	5%	

(1) All with sacral fracture and contralateral crescent fracture of the ilium.

(2) Counting: Dementia, cerebrovascular disease, pulmonary disease, diabetes mellitus, rheumatoid arthritis, known malignancy.

### Surgery

Operation was carried out at a median of 5 days after admission (IQR 2–8.5, 0–26). Fifty-two patients (61%) had additional posterior implants; anterior instability was addressed in 38 patients (Table 2).

**Table 2:** Detailed operative treatment of posterior and anterior pelvis.

	Number	%
**Posterior implants**	85	
TB alone	33	39%
TB + unilateral IS screw	16	19%
TB + bilateral IS screws (3 patients with cement augmentation)	31	36%
TB + anterior plate iliac plate trough 1st window of ilioninguinal approach	4	5%
TB + spinopelvic fixation	1	1%
**Anterior instability**	63	
No anterior stabilization	15	24%
Plate osteosynthesis via modified Stoppa approach	21	33%
Unilateral retrograde transpubic screw	23	37%
Bilateral retrograde transpubic screw	3	5%
Unilateral retrograde transpubic screw + plate osteosynthesis via modified Stoppa approach	1	1%

*TB* trans-sacral bar; *IS* ilio-sacral.

There were no serious vascular or neurologic complications, no fracture progression occurred in the posterior pelvis. Thirteen patients (15.3%) underwent revision surgery related to TB:

- Four patients had revision for a loosened TB (all without additional IS screws and all with delayed presentation, two with persisting anterior instability and one with non-union of the sacral fracture); two of these patients developed peri-implant infection after the revision operation needing operative debridement.
- Four hematomas required operative debridement (one with additional interventional coiling). One infected hematoma was debrided. One peri-implant infection with an epidural abscess was debrided.
- Two TB were removed due to pain (one non-specific pelvic pain, one with pain in S1 dermatome due to malpositioning).
- One malpositioned TB was removed and changed to a transiliac internal fixator.

Revision for TB did not correlate with age (p 0.490), nor with number of comorbidities (p 0.277) or grading of FFP (p 0.105). In two patients, a plate osteosynthesis of the anterior pelvic ring loosened over time and was revised.

Fifteen patients were admitted postoperatively to the ICU for 1 to 4 days (18%). The median total LOS was 18 days (IQR 14–24, 6–92), the postoperative LOS was 12 days (IQR 9–15.5, 3–74).

Medical complications occurred in 24 patients (28%) during hospitalization: 15 urinary tract infections, 4 pneumonias, 4 skin ulcers, 3 deep venous thromboses, one of those had a pulmonary embolism, and one cardiovascular event. Complications correlated with larger total (p 0.003, median 24 vs 17 days) and preoperative LOS (p 0.007, median 4 vs 7 days), there was no correlation with patient’s age (p 0.685), ASA (p 0.744), number of comorbidities (p 0.958), postoperative LOS (p 0.252) or presentation with delay (p 0.268). Mobility and residential status at discharge and FU are depicted in Table 3.

**Table 3:** Mobility and residential status at discharge and time of follow up.

	At discharge		At follow-up		%
**Mobility**					
Mobile on ward	34	40%	Walking without aid	10	18%
Mobile in room	12	14%	Walking with aid	35	64%
Transfer bed—wheelchair	34	40%	Transfer bed—wheelchair	8	14%
Bedridden	4	5%	Bedridden	2	4%
Unknown	1	1%			
**Residential status**					
Home independent with aid	21	25%		46	84%
Nursing home	14	17%		3	5%
Rehabilitation	24	28%		1	2%
Geriatric ward	20	23%		5	9%
Other hospital	5	6%			
Unknown	1	1%			

### Follow-up

**Figure 3:**
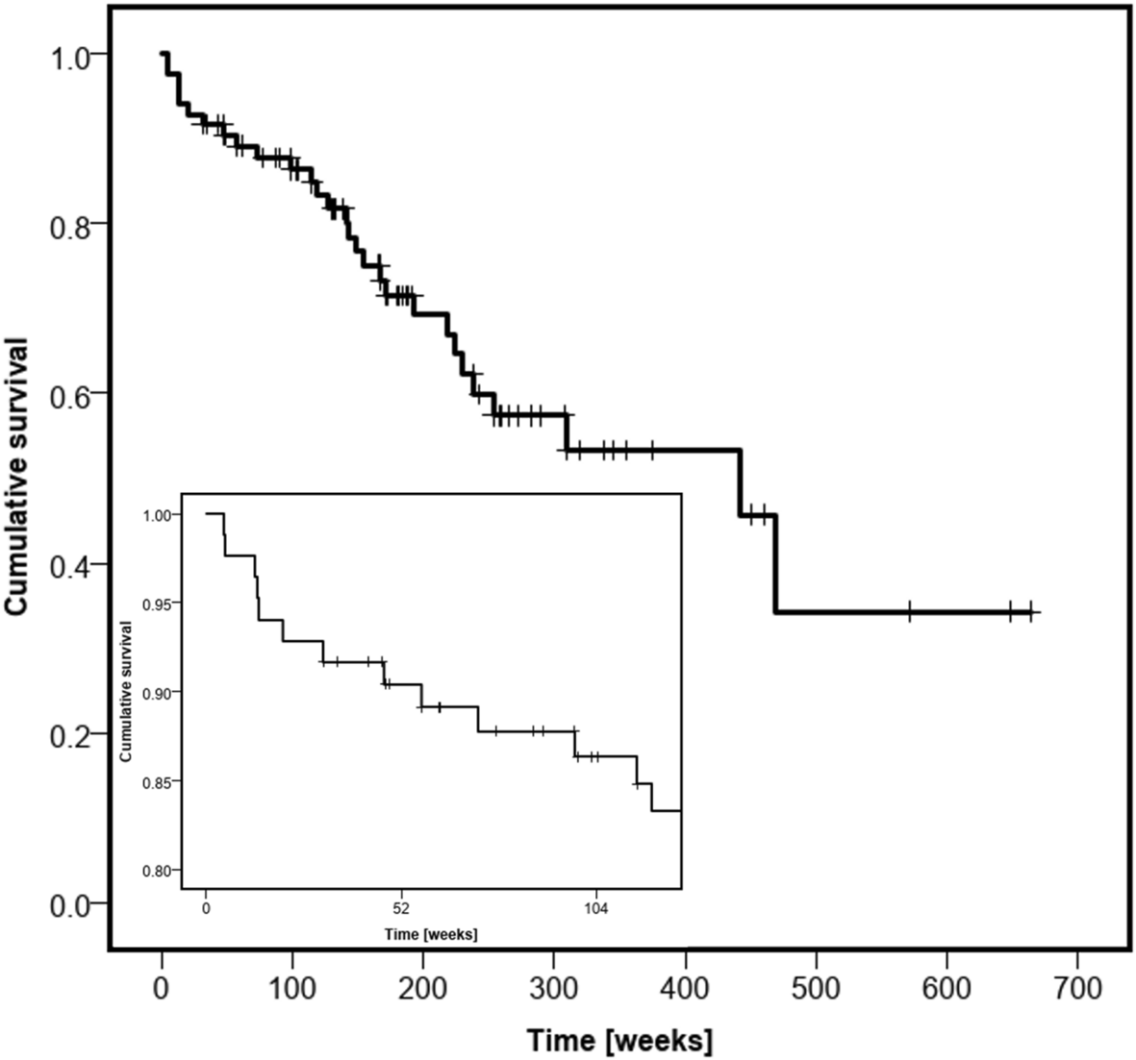
Kaplan–Meier survival analysis. Insert highlights survival in first 2 years of follow-up.

Overall, median FU was 166.9 weeks (IQR 82–259, 2–663). Twenty-nine patients were reported dead (35%) during complete FU (four died later than FU interview), the status of one was unknown (Fig. 1). They died at a mean of 148.8 weeks after admission (± 121, 4.6–469). Cumulative survival rate according to Kaplan–Meier was 90.4% after 1 year, 86.3% after 2 years, and 57.4% after 5 years (Fig. 3).

One-year mortality correlated with a longer total (p 0.006, median 24 vs 17 days) and preoperative LOS (p 0.025, median 10 vs 4 days), however postoperative LOS did not (p 0.337, median 14 vs 12 days). Patients did not differ in age (p 0.94, median 78.0 for dead vs 78.5), ASA score (p 0.62, median 3.0 for dead vs 3.0), number of comorbidities (p 0.47, median 2.0 for dead vs 1.0) or gender (p 0.42, all females). There was a higher risk of 1-year mortality in patients with a medical complication (OR 8.44, 95% CI 1.49–47.70, p 0.006). No statistical difference was found for patients walking at discharge (OR 0.37, 95% CI 0.07–2.02, p 0.234), presenting > 1 week after trauma (OR 1.04, 95% CI 0.19–5.82, p 0.963), or undergoing revision surgery (OR 2.15, 95% CI 0.37–12.50, p 0.387).

Median FU of the 55 living patients was 173.4 weeks (IQR 99–282, 31–663; Table 3). Twenty patients (36%) sustained another osteoporotic fracture during FU at a mean of 122.9 weeks (± 91.8, 5.3–369.7). The mean EQ5D-index was 0.527 (± 0.30, − 0.205 to 1.0). It correlated with mobility (PMS, R 0.660, p < 0.001) and pain (NRS, R − 0.433, p 0.001), there was no correlation with age (R 0.031, p 0.823), ASA (R − 0.108, p 0.433), and number of comorbidities (R 0.017, p 0.903). There was a higher EQ5D with walking at FU (0.60 vs 0.21, p < 0.001) and living at home at FU (0.59 vs 0.20, p < 0.001). No correlation was found to mobility at discharge (walking 0.54 vs 0.51, p 0.722). The median PMS was 5.0 (IQR 3–6, 0–9), it was higher in patients living at home at FU (median 6 vs. 0, p < 0.001). Age (R − 0.237, p 0.081), pain-level (NRS, R 0.027, p 0.844), and walking at discharge from hospital (median 5 for both, p 0.72) did not correlate. Twenty-eight patients (51%) did take some kind of anti-osteoporotic medication at follow-up.

## Discussion

We reported 85 patients suffering from an FFP treated with TB. The 28% medical complication rate and 18 days median LOS represent the frail, multimorbid patient collective. Revision surgery for surgery-related complications was necessary in 15%. There was a high 1-year survival rate of 90%. After a mean follow-up of more than 3 years, 84% of surviving patients were living at home and 82% were walking. However, one-third did suffer another osteoporotic fracture during follow-up.

The patients requiring surgery for FFP are geriatric patients with a median age of 78 years, they were mostly frail, often reported a fall from standing height, and presented with multiple comorbidities. Seventy-two percent had an ASA of 3 or higher, which is more than in previous studies with 49–68%^1,13,24^. Half of the patients had two or more comorbidities. A median LOS of 18 days could be explained by failed trial of conservative treatment, preoperative conditioning, medical complications, or operative revisions. In literature, there is a range of LOS from 4.5 days up to 39 days, probably due to different local medical structures as co-geriatric management^1,25^. Risk factors for prolonged hospital admission include combined posterior and anterior pelvic ring injuries^1^ and operative management^5^.

The golden standard for FFP is conservative treatment. Patients with immobilizing or prolonged pain, displaced fractures or fracture progression^3^ may profit from surgical stabilization. In conservative treatment, the rate of medical complications is 20–53%^1,26,27^. Operatively treated patients tend to have less medical complications (13–28%)^16,24,28,29^. We confirmed lower complication rates with surgery (28%). Longer total and preoperative LOS in hospital correlated with medical complications. This advocates a timely decision for surgery, if necessary.

In our series, we did not have serious vascular or neurologic complications. Operative revision was required in 15% of our patients, other case series mentioned 6–16%^13,16,28,29^. All loosened implants (4.7%) were in patients with delayed presentation and without additional posterior osteosynthesis^13^. This supports a prompt operative intervention if conservative treatment fails. We suppose that bilateral locking with locknuts prevent loosening compared to 9% loosening in trans-sacral fixation using conventional screws. We changed our practice to augment TB with an additional IS screw on the fractured side. This may decrease rotation around the axis of the TB^30^. Less loosening and backing out in IS screw fixation in elderly was shown by using multiple posterior implants^13^. Anterior fixation, whenever possible minimally invasive using retrograde trans-pubic screws^31^ or alternatively using plate osteosynthesis^32^, is important to allow immediate mobilization if there is a concomitant anterior instability. Half of the loosened TB were observed in patients with persistent anterior instability^3^.

We confirmed the low 1-year mortality after surgical treatment of FFP (10–12%)^13,29^ compared to conservative treatment (17–23%)^1,4,6,25,27,33^. Elderly with a pelvic fracture show an increased mortality^4^. The 1-year mortality after surgical treatment of proximal femur fracture is higher with 19–33%^7,8^. This study showed increased mortality at 1 year if a medical complication occurred. Interestingly, there was no correlation to age, ASA, number of comorbidities, or with reduced mobility at discharge.

At a follow-up of more than 3 years, patients were living at home in 84% with 16% being institutionalized at that moment. A similar institutionalization rate of 9–13% 6 months after a pelvic fracture in elderly was observed in an insurance-based study from Germany^2^. Markedly higher institutional rates at follow-up were reported in literature with 25–51%^1,13,25–27^. Only 25% of our patients were primarily discharged home, more than half went to a rehabilitation or geriatric unit. Patients may benefit from more intensive rehabilitation in such institutions after surgical fixation with a high number returning to their home during follow-up. The same may apply for the high rate of 82% mobilization.

The QoL was reduced to a certain extent with an EQ-5D index of 0.53. Normative data in Germany in a cohort of > 75 years was 0.771–0.882^34^. A similarly low index of 0.51 was described in patients with femoral neck fractures (mean age 80 years)^35^ and 0.61 in such with hip fractures (mean age 81 years)^36^, both exhibited significantly lower values at follow up as before the injury. With a median PMS of 5, the mobility was slightly more limited compared to proximal femur fractures with a mean of 5.8 after 12 months^37^.

As medical complications and 1-year mortality were increased in patients with longer total and preoperative LOS, a shorter preoperative time and hence shorter stay at the hospital may be beneficial. In geriatric hip fractures, a delay in surgery increased risk for mortality as well as for complications^38^. A standardized management protocol of analgetic medication and early assisted mobilization may be beneficial together with geriatric co-management.

To our best knowledge, this study represents the largest cohort of patients with trans-sacral fixation as well as operative treatment of FFP. Our study was limited by the large range of FU time with up to 5 years. We suppose a surgery-related difference in mortality and QoL is possible in the first months, however, once the fracture is healed, outcomes may be more dependent on comorbidities. The preoperative quality of life was not assessed, as large follow-up times and increasing age may lead to inconsistent answers. The duration of operation was not evaluated because of different anterior osteosynthesis techniques and change from prone to supine position in case of additional anterior stabilization. The same accounts for intraoperative blood loss. With minimal-invasive technique the blood loss is not collected and thus not measurable. Another limitation is the lack of control group using alternative surgical treatment methods. Although following our protocol to recommend operative treatment for patients with displaced posterior pelvic ring fractures (FFP types 3 and 4) and in non-displaced fractures (FFP type 2) after failed conservative treatment, there may be a selection bias due to the operative expertise in our centre. Suffering from a pelvic fracture after none or a low-energy trauma was defined as fragility fracture, although osteoporosis was not formally assessed by bone mineral density measurement^39^.

## Conclusion

Trans-sacral bar osteosynthesis is a safe and effective technique to mobilize patients with FFP. We recommend a standardized management protocol and, if necessary, timely operative fixation in patients with FFP to lower LOS, increase QoL, and decrease mortality. Posterior augmentation with IS screw and concomitant anterior fixation of instability is recommended.
